# Prederivatives of gamma paraconvex set-valued maps and Pareto optimality conditions for set optimization problems

**DOI:** 10.1186/s13660-017-1519-4

**Published:** 2017-10-02

**Authors:** Hui Huang, Jixian Ning

**Affiliations:** 1 Department of Mathematics, Yunnan University, Cuihu North Road, Kunming, 650091 P.R. China; 2 No.1 High school of Jimo, Culture Road, Jimo, Qindao 266200 P.R. China

**Keywords:** 49J52, 49J53, *γ*-paraconvex set-valued mapping, Pareto minimal solution, set optimization problem

## Abstract

Prederivatives play an important role in the research of set optimization problems. First, we establish several existence theorems of prederivatives for *γ*-paraconvex set-valued mappings in Banach spaces with $\gamma>0$. Then, in terms of prederivatives, we establish both necessary and sufficient conditions for the existence of Pareto minimal solution of set optimization problems.

## Introduction

Let *X* and *Y* be Banach spaces. We say that $G: X\rightrightarrows Y$ is a set-valued mapping if $G(x)$ is a subset of *Y* for all $x\in X$. Set-valued problems occur in many situations, such as control problems, feasibility problems, optimality problems, equilibrium problems and variational inequality problems. A powerful tool dealing with set-valued problems is set-valued analysis. We refer the reader to the references [1–4] for more knowledge about set-valued analysis and its applications.

In a pioneering work [5], Ioffe introduced a notion of prederivative which can be viewed as an extension of Clarke generalized gradient. It is well known that the prederivative is an effective tool in dealing with nondifferentiable mapping of nonsmooth analysis. In contrast with the derivative, the prederivative may not be unique. However, in terms of prederivatives, one can establish an inverse function theorem and implicit theorem and solve nondifferential inclusion problems [6]. In the later publication of Pang [7, 8], and Gaydu, Geoffroy and Jean-Alexis [9], some notions of prederivatives were posed and further studied. In 2016, Gaydu, Geoffroy and Marcelin [10] studied the existence of some kinds of prederivatives of convex set-valued mappings and established necessary and sufficient optimality conditions for the weak minimizers and the strong minimizers of set optimization problems. *γ*-paraconvex set-valued mappings are an extension of convex set-valued mappings, and were studied by some researchers [11, 12]. Moreover, in set optimization problems, Pareto minimizers are more suitable than weak minimizers and strong minimizers in practice [13, 14]. Now, two natural questions are posed. Can we establish some existence results of some kinds of prederivatives for *γ*-paraconvex set-valued mappings? Can we give optimality conditions for the Pareto minimizers of set optimization problems by prederivatives?

In this paper, we firstly establish several existence theorems of prederivatives for *γ*-paraconvex set-valued mappings and cone-*γ*-paraconvex set-valued mappings. Then we establish necessary and sufficient optimality conditions for the Pareto minimizers of set optimization problems in terms of prederivatives.

## Preliminaries

Throughout this paper, unless stated otherwise, we always assume that *X* and *Y* are real Banach spaces and $G:X\rightrightarrows Y$ is a set-valued mapping. The domain of *G* is defined by

$$ \operatorname {Dom}(G)=\bigl\{ x\in X \mid G(x)\neq\emptyset\bigr\} . $$

The graph of *G* is defined by

$$ \operatorname {Gr}(G)=\bigl\{ (x, y)\in X\times Y\mid y\in G(x)\bigr\} . $$

We say that *G* is a closed set-valued mapping if $\operatorname {Gr}(G)$ is a closed subset of $X\times Y$. We say that *G* has convex values if $G(x)$ is a convex subset of *Y* for any $x \in X$. Let Ω be a subset of *X*; we use $\operatorname {cl}(\Omega)$ to denote the closure of Ω, $\operatorname {int}(\Omega)$ to denote the interior of Ω. We use $B_{X}$ and $B_{Y}$ to denote the closed unit ball of *X* and *Y*, respectively. Let $\bar{x}\in X$. We use $N(\bar{x})$ to denote all open neighborhoods of *x̄*. Let $C\subseteq Y$ be a nonempty set. We say that *C* is a cone, if $\lambda c\in C$ for any $c\in C$ and $\lambda\geq0$. We say that *C* is pointed if $C\cap(-C)=\{0\}$. Define $G+C:X\rightrightarrows Y$ as

$$ (G+C) (x):=G(x)+C,\quad \forall x\in X. $$

The following definition is needed in the sequel.

### Definition 2.1

[1]

Let $\Phi :X\rightrightarrows Y$ be a set-valued mapping. We say that Φ is positively homogeneous if $0\in\Phi(0)$ and $\Phi(\lambda x)=\lambda\Phi(x)$, $\forall x \in X$, $\forall \lambda>0$.

### Definition 2.2

[12]

Let $C\subseteq Y$ be a convex cone, $\gamma>0$ and $\eta>0$. We say that *G* is a *C*-*γ*-paraconvex set-valued mapping with modulus *η*, if

$$ \theta G(x)+(1-\theta)G(u)\subseteq G\bigl(\theta x+(1-\theta)u\bigr)+\eta\min \{\theta, 1-\theta\}\Vert x-u\Vert ^{\gamma}B_{Y} +C $$

for all $x , u\in X$, $\theta\in[0, 1]$. We say that *G* is a *γ*-paraconvex set-valued mapping if $C=\{0\}$.

### Remark 2.1

In the special case of $\eta={0}$ and $C=\{0\}$, *C*-*γ*-paraconvex set-valued mappings reduce to convex set-valued mappings.

### Definition 2.3

[15]

We say that *G* is Lipschitz continuous at *x̄* if there exist $l>0$ and $U \in N(\bar{x})$ such that

$$ G(x)\subseteq G\bigl(x'\bigr)+l\bigl\Vert x-x'\bigr\Vert B_{Y}, \quad \forall x, x'\in U. $$

If the above equation holds on $U=\Omega$, then we say that *G* is Lipschitz continuous on Ω.

### Definition 2.4

[10]

Let $\Phi: X\rightrightarrows Y$ be a positively homogeneous set-valued mapping, $\bar{x}\in X$ and $\bar{y}\in G(\bar{x})$.

(i) Φ is called an outer prederivative of *G* at *x̄*, if for any $\delta>0$ there exists $U \in N(\bar{x})$ such that
  $$ G(u)\subseteq G(\bar{x})+\Phi(u-\bar{x})+\delta \Vert u-\bar {x}\Vert B_{Y}, \quad \forall u\in U . $$
(ii) Φ is called a strict prederivative of *G* at *x̄*, if for any $\delta>0$ there exists $U \in N(\bar{x})$ such that
  $$ G(u)\subseteq G\bigl(u'\bigr)+\Phi\bigl(u-u'\bigr)+ \delta\bigl\Vert u-u'\bigr\Vert B_{Y},\quad \forall u, u'\in U. $$
(iii) Φ is called a pseudo strict prederivative of *G* at $(\bar{x}, \bar{y})$, if for any $\delta>0$ there exist $U\in N(\bar {x})$ and $V \in N(\bar{y})$ such that
  $$ G(u)\cap V\subseteq G\bigl(u'\bigr)+\Phi\bigl(u-u' \bigr)+\delta\bigl\Vert u-u'\bigr\Vert B_{Y}, \quad \forall u, u'\in U. $$

## Prederivatives of gamma paraconvex set-valued mappings

In this section, we establish the existence results of pseudo strict prederivatives for *γ*-paraconvex set-valued mappings and strict prederivatives for *C*-*γ*-paraconvex set-valued mappings, respectively.

### Lemma 3.1

[11]

*Let* $G:X\rightrightarrows Y$ *be a closed set*-*valued mapping*, $y_{0}\in G(X)$, $x_{0}\in X$, $\eta>0$, $\delta>0$, $\gamma>0$, $G^{-1}$ *be a* *γ*-*paraconvex set*-*valued mapping with modulus* *r*, *and* $y_{0}+\eta B_{Y}\subseteq G(x_{0}+\delta B_{X})$. *Let* $\eta_{1}>0$, $\eta_{2}>0$ *with* $\eta_{1}+\eta_{2}=\eta$. *Then*, *for each* $y\in y_{0}+\eta_{1}B_{Y}$,

$$ d\bigl(x, G^{-1}(y)\bigr)\leq\frac{d(y, G(x))}{\eta_{2}}\bigl(\delta +r\eta ^{\gamma}_{2}+\Vert x-x_{0}\Vert \bigr),\quad \forall x \in X. $$

### Theorem 3.1

*Let* $\eta>0$, $\delta>0$, $r>0$, $\gamma>0$, $G:X\rightrightarrows Y$ *be a closed* *γ*-*paraconvex set*-*valued mapping with modulus* *r*, $(\bar{x}, \bar{y})\in \operatorname {Gr}(G)$ *and* $\bar{x}+\eta B_{X}\subseteq G^{-1}(\bar{y}+\delta B_{Y})$. *Then* *G* *has a pseudo strict prederivative* Φ *at* $(\bar{x}, \bar{y})$ *with* $\Phi(\cdot )=L\Vert \cdot \Vert B_{Y}$, *where* $L=(\delta+r(\frac{\eta }{2})^{\gamma}+\frac{\eta}{2})\frac{2}{\eta}>0$.

### Proof

Clearly, $G^{-1}$ is a closed set-valued mapping since *G* is a closed set-valued mapping. Since *G* is a *γ*-paraconvex set-mapping and $\bar{x}+\eta B_{X}\subseteq G^{-1}(\bar{y}+\delta B_{Y})$, it follows from Lemma 3.1 that, for each $x\in\bar{x}+\frac{\eta}{2}B_{X}$,

$$ d\bigl(y, G(x)\bigr)\leq\frac{d(x, G^{-1}(y))}{\frac{\eta}{2}} \biggl( \delta+r \biggl( \frac{\eta}{2} \biggr) ^{\gamma}+\Vert y-\bar{y} \Vert \biggr) ,\quad \forall y\in Y . $$

Then, for any $y\in\bar{y}+\frac{\eta}{2} B_{Y}$,

$$ d\bigl(y, G(x)\bigr)\leq\biggl( \delta+r \biggl( \frac{\eta}{2} \biggr) ^{\gamma}+\frac{\eta}{2} \biggr) \frac{2}{\eta}{d\bigl(x, G^{-1}(y)\bigr)}. $$

Let $L:=(\delta+r(\frac{\eta}{2})^{\gamma}+\frac{\eta}{2})\frac {2}{\eta}$. We have

$$ d\bigl(y, G(x)\bigr)\leq Ld\bigl(x, G^{-1}(y)\bigr), \quad \forall x\in \bar{x}+\frac{\eta}{2}B_{X}, \forall y\in\bar{y}+ \frac{\eta}{2}B_{Y}. $$

This implies that

$$ y\in G(x)+Ld\bigl(x, G^{-1}(y)\bigr)B_{Y}, \quad \forall x\in \bar{x}+\frac{\eta}{2}B_{X}, \forall y\in\bar{y}+ \frac{\eta}{2}B_{Y}. $$

Then, for any $\tilde{\delta}>0$,

$$ G\bigl(x'\bigr)\cap\biggl(\bar{y}+\frac{\eta}{2}B_{Y} \biggr)\subseteq G(x)+L\bigl\Vert x-x'\bigr\Vert B_{Y}+ \tilde{\delta}\bigl\Vert x-x'\bigr\Vert B_{Y}, \quad \forall x, x'\in\bar{x}+\frac{\eta}{2}B_{X}. $$

Therefore $\Phi(\cdot):=L\Vert \cdot \Vert B_{Y}$ is a pseudo strict prederivative of *G* at $(\bar{x}, \bar{y})$. □

### Remark 3.1

Theorem 3.1 extends [10, Theorem 3.3] from convex set-valued mappings to *γ*-paraconvex set-valued mappings. Recall [16] that *G* is said to be open at $(\bar{x}, \bar{y})\in \operatorname {Gr}(G)$ if $G(U)$ is a neighborhood of *ȳ* for every neighborhood *U* of *x̄*. The assumption $\bar{x}+\eta B_{X}\subseteq G^{-1}(\bar{y}+\delta B_{Y})$ means that $G^{-1}(\bar{y}+\delta B_{Y})$ is a neighborhood of *x̄*, which is very close to the openness property of $G^{-1}$ at $(\bar{x}, \bar{y})$. However, the coefficients *δ* and *η* are fixed in our assumption.

### Lemma 3.2

[17, Lemma 1]

*Let* *A*, *B* *and* *D* *are subsets of* *X*. *If* *B* *is a closed convex set*, *D* *is a bounded set and* $A+D\subseteq B+D$, *then* $A\subseteq B$.

### Theorem 3.2

*Let* $C\subseteq Y$ *be a nonempty closed convex cone*, $\eta>0$, $\gamma\geq1$, $r>0$, $\alpha>0$, $G:X\rightrightarrows Y$ *be a* *C*-*γ*-*paraconvex set*-*valued mapping with modulus* *r*, $\bar{x}+\alpha B_{X}\subseteq \operatorname {Dom}(G)$. *Assume that*

3.1 $$ G(x)=A(x)+E(x), \quad \forall x\in\bar{x}+\alpha B_{X}, $$

*where* $A(x)$ *is a subset of* *Y* *and* $E(x)$ *is a convex set with* $A(x)\subseteq\eta B_{Y}$ *and* $0\in E(x)\subseteq C$. *Let* $\Phi:X\rightrightarrows Y$ *be defined by*

$$ \Phi(x)= \biggl( \frac{16\eta}{\alpha}+r\biggl(\frac{3\alpha}{4} \biggr)^{\gamma-1} \biggr) \Vert x\Vert B_{Y}, \quad \forall x\in X. $$

*Then the following conclusions hold*:

(i) $G+C$ *is Lipschitz on* $\bar{x}+\frac{\alpha}{4}B_{X}$ *with modulus* $\frac{16\eta}{\alpha}+r(\frac{3\alpha}{4})^{\gamma-1}$;
(ii) Φ *is a strict prederivative of* $G+C$ *at each* $x\in\bar {x}+\frac{\alpha}{8}B_{X}$;
(iii) $\Phi+C$ *is a strict prederivative of* *G* *at each* $x\in\bar {x}+\frac{\alpha}{8}B_{X}$.

### Proof

(i) Take $x_{1}, x_{2}\in\bar{x}+\frac{\alpha}{4}B_{X}$ with $x_{1}\neq x_{2}$. Let $\lambda:=\frac{\alpha}{4}$ and define

$$ z:=x_{1}-\lambda\frac{x_{2}-x_{1}}{\Vert x_{2}-x_{1}\Vert }. $$

Then

$$ x_{1}=\frac{\lambda}{ \Vert x_{2}-x_{1}\Vert +\lambda}x_{2}+\frac {\Vert x_{2}-x_{1}\Vert }{\Vert x_{2}-x_{1}\Vert +\lambda}z. $$

Let $\theta:=\frac{\Vert x_{2}-x_{1}\Vert }{\Vert x_{2}-x_{1}\Vert +\lambda}$. Then $\theta\in(0,1)$ and $x_{1}=(1-\theta)x_{2}+\theta z$. Since $z-x_{2}=\frac{x_{1}-x_{2}}{\theta}$ and $\gamma\geq1$, we have

3.2 $$ \begin{aligned}[b] & \min\{\theta, 1-\theta\}\Vert x_{2}-z\Vert ^{\gamma} \\ &\quad = \min\{\theta, 1-\theta\}\frac{\Vert x_{1}-x_{2}\Vert ^{\gamma}}{\theta^{\gamma}} \leq\theta\frac{\Vert x_{1}-x_{2}\Vert ^{\gamma}}{\theta^{\gamma}} \\ &\quad =\frac{\Vert x_{1}-x_{2}\Vert ^{\gamma}}{\theta^{\gamma-1}} = \frac{\Vert x_{1}-x_{2}\Vert ^{\gamma}}{ ( \frac{\Vert x_{1}-x_{2}\Vert }{\Vert x_{1}-x_{2}\Vert +\lambda} ) ^{\gamma-1}} \\ &\quad \leq\bigl(\Vert x_{1}-x_{2}\Vert +\lambda \bigr)^{\gamma-1}\Vert x_{1}-x_{2}\Vert \\ &\quad \leq\biggl( \frac{\alpha}{2}+\frac{\alpha}{4} \biggr) ^{\gamma -1}\Vert x_{1}-x_{2}\Vert =\biggl(\frac{3\alpha}{4} \biggr)^{\gamma-1}\Vert x_{1}-x_{2}\Vert . \end{aligned} $$

Since *G* is a *C*-*γ*-paraconvex set-valued mapping with modulus *r*, it is easy to verify that $G+C$ is a *γ*-paraconvex set-valued mapping with modulus *r*. Taking into account inequality (3.2), we have

3.3 $$ \begin{aligned}[b] & (1-\theta) (G+C) (x_{2})+\theta(G+C) (z) \\ &\quad \subseteq(G+C) (x_{1})+r\min\{\theta, 1-\theta\}\Vert x_{2}-z\Vert ^{\gamma}B_{Y} \\ &\quad \subseteq(G+C) (x_{1})+r\biggl(\frac{3\alpha}{4} \biggr)^{\gamma-1}\Vert x_{1}-x_{2}\Vert B_{Y}. \end{aligned} $$

Due to the convexity of $(G+C)(x)$ for each $x\in X$, we have

3.4 $$ (1-\theta) (G+C) (x_{2})+\theta(G+C) (x_{2})=(G+C) (x_{2}). $$

Adding $\theta(G+C)(x_{2})$ on both sides of equation (3.3), and using (3.4), we get

$$ (G+C) (x_{2})+\theta(G+C) (z)\subseteq(G+C) (x_{1})+\theta (G+C) (x_{2})+ r\biggl(\frac{3\alpha}{4}\biggr)^{\gamma-1}\Vert x_{1}-x_{2}\Vert B_{Y}. $$

Clearly, $C+\theta C=C+C=2C=C$ since *C* is a convex cone. Therefore, the above equation can be rewritten as

3.5 $$ G(x_{2})+\theta G(z)+C\subseteq G(x_{1})+\theta G(x_{2})+C+r\biggl(\frac{3\alpha}{4}\biggr)^{\gamma-1}\Vert x_{1}-x_{2}\Vert B_{Y}. $$

Since $A(x)\subseteq\eta B_{Y}$ for any $x\in\bar{x}+\alpha B_{X}$, we obtain

3.6 $$ A(x)\subseteq A\bigl(x'\bigr)+2\eta B_{Y}, \quad \forall x, x'\in\bar{x}+\alpha B_{X}. $$

Next, we show that $z\in\bar{x}+\alpha B_{X}$. Since $x_{1}=(1-\theta )x_{2}+\theta z$, we have

$$\begin{aligned} \Vert z-\bar{x}\Vert &\leq \Vert z-x_{2}\Vert +\Vert x_{2}-\bar{x}\Vert \\ &\leq\frac{\Vert x_{1}-x_{2}\Vert }{\theta}+\frac{\alpha}{4} \\ &= \Vert x_{1}-x_{2}\Vert +\lambda+\frac{\alpha}{4} \\ &\leq \Vert x_{1}-\bar{x}\Vert +\Vert \bar{x}-x_{2} \Vert +\frac{\alpha}{4}+\frac{\alpha}{4} \\ &\leq\frac{\alpha}{4}+\frac{\alpha}{4}+\frac{\alpha}{2}=\alpha. \end{aligned}$$

Therefore $z\in\bar{x}+\alpha B_{X}$. As $x_{2}, z\in\bar{x}+\alpha B_{X}$, combined with (3.6), we have

$$ A(x_{2})\subseteq A(z)+2\eta B_{Y}. $$

By the assumption (3.1), $G(z)=A(z)+E(z)$, $G(x_{2})=A(x_{2})+E(x_{2})$, it follows from (3.5) that

3.7 $$ \begin{aligned}[b] & G(x_{2})+\theta\bigl(A(z)+E(z)\bigr)+C \\ &\quad \subseteq G(x_{1})+\theta\bigl(A(x_{2})+E(x_{2}) \bigr)+C+r\biggl(\frac{3\alpha}{4}\biggr)^{\gamma-1}\Vert x_{1}-x_{2}\Vert B_{Y} \\ &\quad \subseteq G(x_{1})+\theta\bigl(A(z)+2\eta B_{Y}\bigr)+ \theta E(x_{2})+C+r\biggl(\frac{3\alpha}{4}\biggr)^{\gamma-1}\Vert x_{1}-x_{2}\Vert B_{Y}. \end{aligned} $$

Since $0\in E(x_{2})\subseteq C$ and $0\in E(z)\subseteq C$, we have

$$ \theta E(x_{2})+C=C, \quad\quad \theta E(z)+C=C. $$

Equation (3.7) yields

3.8 $$ \begin{aligned}[b] & G(x_{2})+C +\theta A(z) \\ &\quad \subseteq G(x_{1})+\theta A(z)+2\eta\theta B_{Y}+C+r \biggl(\frac{3\alpha}{4}\biggr)^{\gamma-1}\Vert x_{1}-x_{2} \Vert B_{Y} \\ &\quad \subseteq \operatorname {cl}\biggl(G(x_{1})+2\eta\theta B_{Y}+C+r\biggl( \frac{3\alpha}{4}\biggr)^{\gamma-1}\Vert x_{1}-x_{2} \Vert B_{Y}\biggr)+\theta A(z). \end{aligned} $$

Since $\operatorname {cl}(G(x_{1})+2\eta\theta B_{Y}+C+r(\frac{3\alpha}{4})^{\gamma -1}\Vert x_{1}-x_{2}\Vert B_{Y})$ is a closed convex set and $A(z)$ is a bounded set, it follows from Lemma 3.2 and (3.8) that

3.9 $$ \begin{aligned}[b] G(x_{2})+C &\subseteq \operatorname {cl}\biggl(G(x_{1})+2 \eta\theta B_{Y}+C+r\biggl(\frac{3\alpha}{4}\biggr)^{\gamma-1} \Vert x_{1}-x_{2}\Vert B_{Y}\biggr) \\ &\subseteq G(x_{1})+4\eta\theta B_{Y}+C+r\biggl( \frac{3\alpha}{4}\biggr)^{\gamma-1}\Vert x_{1}-x_{2} \Vert B_{Y} \\ &=G(x_{1})+C+\biggl(4\eta\theta+r \biggl(\frac{3\alpha}{4} \biggr)^{\gamma-1}\Vert x_{1}-x_{2}\Vert \biggr)B_{Y} \\ &=G(x_{1})+C+ \biggl( \frac{4\eta \Vert x_{2}-x_{1}\Vert }{\Vert x_{2}-x_{1}\Vert +\lambda}+r\biggl(\frac{3\alpha}{4} \biggr)^{\gamma-1}\Vert x_{1}-x_{2}\Vert \biggr) B_{Y} \\ &\subseteq G(x_{1})+C+ \biggl( \frac{4\eta}{\lambda}+r\biggl( \frac{3\alpha}{4}\biggr)^{\gamma-1} \biggr) \Vert x_{2}-x_{1} \Vert B_{Y} \\ &\subseteq G(x_{1})+C+ \biggl( \frac{16\eta}{\alpha}+r\biggl( \frac{3\alpha}{4}\biggr)^{\gamma-1} \biggr) \Vert x_{2}-x_{1} \Vert B_{Y}, \end{aligned} $$

where the last inequality holds since $\lambda=\frac{\alpha}{4}$. Therefore, $G+C$ is Lipschitz with modulus $\frac{16\eta}{\alpha }+r(\frac{3\alpha}{4})^{\gamma-1}$ on $\bar{x}+\frac{\alpha}{4}B_{X}$ since $x_{1}$ and $x_{2}$ are two arbitrary elements of $\bar{x}+\frac{\alpha}{4}B_{X}$.

(ii) Let $\Phi: X\rightrightarrows Y$ be defined by

3.10 $$ \Phi(x)= \biggl( \frac{16\eta}{\alpha}+r\biggl(\frac{3\alpha}{4} \biggr)^{\gamma-1} \biggr) \Vert x\Vert B_{Y}, \quad \forall x\in X. $$

Clearly, Φ is a positively homogeneous mapping with bounded closed values. By (3.9), we get

3.11 $$ G(x_{2})+C\subseteq G(x_{1})+C+\Phi(x_{2}-x_{1})+ \delta \Vert x_{2}-x_{1}\Vert B_{Y}, \quad \forall x_{1}, x_{2}\in\bar{x}+\frac{\alpha}{4}B_{X} $$

for any $\delta>0$. This implies that Φ is a strict prederivative of $G+C$ at each $x\in\bar{x}+\frac{\alpha}{8}B_{X}$.

(iii) Since *C* is a cone, it follows from (3.10) that $0\in (\Phi+C)(0)$, and for any $t>0$ and $x\in X$,

$$ (\Phi+C) (tx)=t\Phi(x)+C=t(\Phi+C) (x), $$

and hence $\Phi+C$ is positively homogeneous. Let $\tilde{x}\in\bar{x}+\frac{\alpha}{8}B_{X}$. Then there exists $\tilde{r}>0$ such that $\tilde{x}+\tilde{r}B_{X}\subseteq\bar{x}+\frac{\alpha}{4}B_{X}$. Since $0\in C$, it follows from (3.11) that for any $\delta>0$,

$$ G(x_{2})\subseteq G(x_{1})+(\Phi+C) (x_{2}-x_{1})+ \delta \Vert x_{2}-x_{1}\Vert B_{Y}, \quad \forall x_{1}, x_{2}\in\tilde{x}+\tilde{r}B_{X}. $$

Therefore, $\Phi+C$ is a strict prederivative of *G* at *x̃*. □

### Remark 3.2

In [10, Theorem 3.8], Gaydu, Geoffroy and Marcelin proved the following result. Let *Y* be a finite dimensional Banach space, $C\subseteq Y$ be a nonempty closed convex cone, $G:X\rightrightarrows Y$ be a *C*-convex set-valued mapping, $\bar {x}\in \operatorname {int}(\operatorname {dom}(G))$. Assume that there exist $\alpha>0$ and $\eta>0$ such that $G(x)+\operatorname {cl}(C)$ is a closed set and $G(x)\subseteq\eta B_{Y}$ for all $x\in\bar{x}+\alpha B_{X}$. Then there exists $U\in N(\bar{x})$ such that

(i) $G+C$ is Lipschitz on *U*;
(ii) there exists a positively homogeneous mapping $\Phi: X\rightrightarrows Y$ with bounded closed values such that Φ is a strict prederivative of $G+C$ at each $x\in U$;
(iii) $\Phi+C$ is a strict prederivative of *G* at each $x\in U$.

In contrast with [10, Theorem 3.8], Theorem 3.2 has some improvements. Firstly, we extend *Y* from finite dimensional spaces to general Banach spaces. Secondly, we extend *G* from *C*-convex set-valued mappings to *C*-*γ*-paraconvex set-valued mappings. Thirdly, we do not need the boundedness of $G(x)$.

In the following, we give an example to illustrate Theorem 3.2.

### Example 3.1

Let $X=Y=\mathbb{R}$, $C=\mathbb {R}_{+}$, $G: \mathbb{R}\rightrightarrows\mathbb{R}$ be defined by

$$ G(x)=[\bigl\vert \vert x\vert -1\bigr\vert ,+\infty), \quad \forall x\in \mathbb{R}. $$

It follows from [12, Example 3.1] that *G* is a *C*-1-paraconvex set-valued mapping with modulus 1, but not a *C*-convex set-valued mapping. Take $\bar{x}=0$, $A(x)=\vert \vert x\vert -1\vert $ and $E(x)=\mathbb{R}_{+}$. Then

$$ G(x)=A(x)+E(x), \quad \forall x\in\bar{x}+B_{X} $$

with $A(x)\subseteq[-1,1]$ for all $x\in\bar{x}+B_{X}$. All conditions of Theorem 3.2 are justified. By Theorem 3.2, $G+C$ is Lipschitz on $\bar{x}+\frac{1}{4}B_{X}$ with modulus 17, and $\Phi(\cdot)=17\vert \cdot \vert B_{Y}$ satisfies (ii) and (iii) of Theorem 3.2 on $\bar{x}+\frac{1}{8}B_{X}$.

### Corollary 3.1

*Let* $C\subseteq Y$ *be a nonempty closed convex cone*, $\eta>0$, $\gamma\geq1$, $r>0$, $\alpha>0$, $G:X\rightrightarrows Y$ *be a* *C*-*γ*-*paraconvex set*-*valued mapping with modulus* *r* *and* $\bar{x}+\alpha B_{X}\subseteq \operatorname {Dom}(G)$. *Assume that*

$$ G(x)\subseteq\eta B_{Y}, \quad \forall x \in\bar{x}+\alpha B_{X}. $$

*Let* $\Phi:X\rightrightarrows Y$ *be defined by*

$$ \Phi(x)= \biggl( \frac{16\eta}{\alpha}+r\biggl(\frac{3\alpha}{4} \biggr)^{\gamma-1} \biggr) \Vert x\Vert B_{Y}, \quad \forall x\in X. $$

*Then the following conclusions hold*:

(i) $G+C$ *is Lipschitz on* $\bar{x}+\frac{\alpha}{4}B_{X}$ *with modulus* $\frac{16\eta}{\alpha}+r(\frac{3\alpha}{4})^{\gamma-1}$;
(ii) Φ *is a strict prederivative of* $G+C$ *at each* $x\in\bar {x}+\frac{\alpha}{8}B_{X}$;
(iii) $\Phi+C$ *is a strict prederivative of* *G* *at each* $x\in\bar {x}+\frac{\alpha}{8}B_{X}$.

### Proof

Take $A(x)=G(x)$, $E(x)=\{0\}$ for all $x\in X$ in Theorem 3.2. Then the conclusions follow from Theorem 3.2 directly. □

## Pareto minimizer and prederivative

In this section, we always assume that *C* is a pointed closed convex cone of *Y*. Consider the following set optimization problem:

$$ (\mathrm{SP}) :\quad \textstyle\begin{cases} \min_{C} & G(x),\\ \mbox{s.t.} & x\in\Omega, \end{cases} $$

where Ω is a nonempty closed subset of *X* with $\operatorname {Dom}(G)\cap \Omega\neq\emptyset$.

### Definition 4.1

[13]

We say that $(\bar{u}, \bar {v})\in \operatorname {Gr}(G)$ is a Pareto minimizer of the optimization problem (SP), if $\bar{u}\in\Omega$ and $(\bar{v}-C)\cap G(\Omega)=\{\bar {v}\}$.

First, we establish a necessary condition for Pareto minimizers of the optimization problem (SP).

### Theorem 4.1

*Let* $\bar{u}\in \operatorname {int}(\Omega)$, $(\bar{u}, \bar{v})\in \operatorname {Gr}(G)$. *Suppose that* Φ *is a pseudo strict prederivative of* *G* *at* $(\bar {u},\bar{v})$ *and* $(\bar{u}, \bar{v})$ *is a Pareto minimizer of the optimization problem* (*SP*). *Then*, *for any* $\delta>0$ *and* $u\in\Omega$,

4.1 $$ \Phi\bigl(-(u-\bar{u})\bigr)+\delta \Vert u-\bar{u}\Vert B_{Y} \nsubseteq C\setminus\{0\}. $$

### Proof

Let $(\bar{u}, \bar{v})$ be a Pareto minimizer of the optimization problem (SP). Suppose that the conclusion is not true. Then there exist $\delta_{0}>0$ and $u_{0}\in\Omega$ such that

4.2 $$ \Phi\bigl(-(u_{0}-\bar{u})\bigr)+\delta_{0}\Vert u_{0}-\bar{u}\Vert B_{Y} \subseteq C\setminus\{0\}. $$

Since Φ is a pseudo strict prederivative of *G* at $(\bar{u},\bar {v})$, there exist $\eta_{1}>0$ and $\eta_{2}>0$ such that

$$ G(u)\cap(\bar{v}+\eta_{2}B_{Y})\subseteq G(\tilde{u})+\Phi (u-\tilde{u})+\delta_{0}\Vert u-\tilde{u}\Vert B_{Y}, \quad \forall u, \tilde{u}\in\bar{u}+\eta_{1}B_{X}. $$

Choose $\theta>0$ such that $\bar{u}+\theta(u_{0}-\bar{u})\in(\bar{u}+\eta_{1}B_{X})\cap\Omega$. By the above equation, we have

$$ G(\bar{u})\cap(\bar{v}+\eta_{2}B_{Y})\subseteq G\bigl( \bar{u}+\theta(u_{0}-\bar{u})\bigr)+\Phi\bigl(-\theta (u_{0}-\bar{u})\bigr)+\delta_{0}\bigl\Vert \theta (u_{0}-\bar{u})\bigr\Vert B_{Y}. $$

Since $\bar{v}\in G(\bar{u})\cap(\bar{v}+\eta_{2}B_{Y}) $, the above equation implies that there exists $\hat{v}\in G(\bar{u}+\theta(u_{0}-\bar{u}))$ such that

$$ \bar{v}-\hat{v}\in\Phi\bigl(-\theta(u_{0}-\bar{u})\bigr)+\delta _{0}\bigl\Vert \theta(u_{0}-\bar{u})\bigr\Vert B_{Y}. $$

Since Φ is positively homogeneous, we get

$$ \theta^{-1}(\bar{v}-\hat{v})\in\Phi\bigl(-(u_{0}-\bar{u}) \bigr)+\delta_{0}\Vert u_{0}-\bar{u}\Vert B_{Y} . $$

Combined with (4.2), we have $\bar{v}-\hat{v}\in C\setminus\{ 0\}$. Noting that $\hat{v}\in G(\bar{u}+\theta(u_{0}-\bar{u}))\subseteq G(\Omega)$, we have

$$ \hat{v}\in\bigl(\bar{v}-C\setminus\{0\}\bigr)\cap G(\Omega). $$

This contradicts the assumption that $(\bar{u}, \bar{v})$ is a Pareto minimizer of the optimization problem (SP). Therefore (4.1) holds. □

### Definition 4.2

[18]

Let $G:X\rightrightarrows Y$ be a set-valued mapping, $\bar{u}\in \operatorname {Dom}(G)$. We say that *G* is *C*-starshaped at *ū*, if for any $u\in X$, $\theta\in[0, 1]$,

$$ (1-\theta) G(\bar{u})+\theta G(u)\subseteq G\bigl((1-\theta)\bar {u}+\theta u \bigr)+C. $$

The following theorem provides a sufficient optimality condition for a Pareto minimizer of the optimization problem (SP).

### Theorem 4.2

*Let* $\bar{u}\in\Omega$, Φ *be an outer prederivative of* *G* *at* *ū*, $\bar{v}\in G(\bar{u})$, $G(\bar{u})\subseteq\bar{v}+C$ *and* *G* *be* *C*-*starshaped at* *ū*. *If there exist* $\delta>0$ *and* $\eta>0$ *such that*

4.3 $$ \bigl(\Phi(u-\bar{u})+\delta \Vert u-\bar{u}\Vert B_{Y}\bigr)\cap (-C)\subseteq\{0\} $$

*for all* $u\in\bar{u}+\eta B_{X}$, *then* $(\bar{u}, \bar{v})$ *is a Pareto minimizer of the optimization problem* (*SP*).

### Proof

Let $u\in\Omega$. Since Φ is an outer prederivative of *G* at *ū*, for the given *δ* in the assumption, there exists $\bar{\eta}\in(0, \eta)$ such that

4.4 $$ G(\tilde{u})\subseteq G(\bar{u})+\Phi(\tilde{u}-\bar{u})+\delta \Vert \tilde{u}-\bar{u}\Vert B_{Y}, \quad \forall\tilde{u}\in\bar{u}+\bar{\eta }B_{X}. $$

Choose $\theta\in(0, 1)$ such that $(1-\theta)\bar{u}+\theta u\in \bar{u}+\bar{\eta}B_{X}$. Since *G* is *C*-starshaped at *ū*, we have

4.5 $$ (1-\theta)G(\bar{u})+\theta G(u)\subseteq G\bigl((1-\theta)\bar {u}+\theta u \bigr)+C. $$

Combined (4.4) with (4.5), we get

$$ \begin{aligned} & (1-\theta)G(\bar{u})+\theta G(u) \\ &\quad \subseteq G(\bar{u})+\Phi\bigl((1-\theta)\bar{u}+\theta u-\bar {u}\bigr)+ \delta\bigl\Vert (1-\theta)\bar{u}+\theta u-\bar{u}\bigr\Vert B_{Y}+C \\ &\quad =G(\bar{u})+\Phi\bigl(\theta(u-\bar{u})\bigr)+\delta\bigl\Vert \theta(u- \bar{u})\bigr\Vert B_{Y}+C. \end{aligned} $$

As $G(\bar{u})\subseteq\bar{v}+C$, we get

$$ \begin{aligned} (1-\theta)\bar{v}+\theta G(u)&\subseteq(1-\theta )G(\bar{u})+ \theta G(u) \\ &\subseteq G(\bar{u})+\Phi\bigl(\theta(u-\bar{u})\bigr)+\delta\bigl\Vert \theta(u-\bar{u})\bigr\Vert B_{Y}+C \\ &\subseteq\bar{v}+\Phi\bigl(\theta(u-\bar{u})\bigr)+\delta\bigl\Vert \theta(u-\bar{u})\bigr\Vert B_{Y}+C, \end{aligned} $$

that is,

4.6 $$ \theta\bigl(G(u)-\bar{v}\bigr)\subseteq\Phi\bigl(\theta(u-\bar {u})\bigr)+ \delta\bigl\Vert \theta(u-\bar{u})\bigr\Vert B_{Y}+C. $$

Since $(1-\theta)\bar{u}+\theta u\in\bar{u}+\bar{\eta} B_{X}$, with (4.3), we have

$$ \bigl(\Phi\bigl(\theta(u-\bar{u})\bigr)+\delta\bigl\Vert \theta (u-\bar{u}) \bigr\Vert B_{Y}\bigr)\cap(-C)\subseteq\{0\}. $$

It follows from (4.6) that

$$ \bigl( G(u)-\bar{v}\bigr)\cap(-C)\subseteq\{0\}. $$

Therefore, $(\bar{u}, \bar{v})$ is a Pareto minimizer of the optimization problem (SP) since *u* is an arbitrary element of Ω. □

### Remark 4.1

In [10], Gaydu, Geoffroy and Marcelin established necessary condition and sufficient conditions for the weak minimizers and the strong minimizers of the optimization problem (SP). It is well known that each strong minimizer is a Pareto minimizer and each Pareto minimizer is a weak minimizer, but the converses are not true.

In the following, we give an example to illustrate Theorem 4.2.

### Example 4.1

In (SP), let $X=\mathbb{R}$, $Y=\mathbb {R}^{2}$, $C=\mathbb{R}^{2}_{+}$, $\Omega=(-\infty, 0]$, $G: X\rightrightarrows Y$ be defined by

$$ G(u)= \textstyle\begin{cases} \{(t_{1}, t_{2}) \mid t_{1}\geq u, t_{2}\geq-u\}, &u\leq0,\\ \emptyset, & u>0. \end{cases} $$

Take $\bar{u}=0$, $\bar{v}=(0,0)\in G(\bar{u})$ and $\delta=\frac {1}{2}$. Then $G(\bar{u})\subseteq\bar{v}+\mathbb{R}^{2}_{+}$. It is easy to verify that *G* is *C*-starshaped at *ū*, $\Phi =(1,-1)$ is an outer prederivative of *G* at *ū* and

$$ \begin{aligned} & \bigl(\Phi(u-\bar{u})+\delta \Vert u-\bar{u}\Vert B_{\mathbb{R}^{2}}\bigr)\cap\bigl(-\mathbb{R}_{+}^{2}\bigr) \\ &\quad =\biggl((u, -u)+\frac{1}{2}\vert u\vert B_{\mathbb {R}^{2}}\biggr)\cap \bigl(-\mathbb{R}_{+}^{2}\bigr)\subseteq\bigl\{ (0,0)\bigr\} ,\quad \forall u\in\bar{u}+\frac{1}{2}B_{X}= \biggl[ - \frac{1}{2},\frac{1}{2} \biggr] . \end{aligned} $$

All conditions of Theorem 4.2 are verified. Therefore, $(\bar {u}, \bar{v})$ is a Pareto minimizer of the optimization problem (SP).

## Conclusion

In this paper, we establish two existence theorems of prederivatives for *γ*-paraconvex set-valued mappings, and give optimality conditions for the Pareto minimizers of set optimization problems. These results improve the corresponding one obtained in [10]. Moreover, the coefficients in Theorems 3.1 and 3.2 can be calculated. Theorems 3.1 and 3.2 give sufficient conditions for the existence of Φ of Theorem 4.1.
